# Mini-review: red seaweed *Hydropuntia edulis* and agar derivatives for food, medicinal and agriculture applications

**DOI:** 10.7717/peerj.20393

**Published:** 2026-01-30

**Authors:** Tracy Saptu, Kaiser Mahmood, Wan-Teng Leong, Mohd Fakhrulddin Ismail, Irina Harun, Uthumporn Utra, Shahrul Razid Sarbini, Ahmad Hussaini, Muta Harah Zakaria, Yus Aniza Yusof, Shiamala Devi Ramaiya, Hanisah Kamilah

**Affiliations:** 1 Department of Crop Science, Faculty of Agricultural and Forestry Sciences, Universiti Putra Malaysia, Sarawak Campus, Bintulu, Sarawak, Malaysia; 2 Food Formulation and Processing, Halal Products Research Institute, Universiti Putra Malaysia, Serdang, Malaysia; 3 International Institute of Aquaculture and Aquatic Sciences (I-AQUAS), Universiti Putra Malaysia, Port Dickson, Malaysia; 4 Department of Environment, Faculty of Forestry and Environment, Universiti Putra Malaysia, Serdang, Malaysia; 5 Food Technology Department, School of Industrial Technology, Universiti Sains Malaysia, Pulau Pinang, Malaysia; 6 Halal Healthcare and Wellness, Halal Products Research Institute, Universiti Putra Malaysia, Serdang, Selangor, Malaysia; 7 Faculty of Resource Science and Technology, Universiti Malaysia Sarawak, Kuching, Malaysia; 8 Department of Aquaculture, Faculty of Agriculture, Universiti Putra Malaysia, Serdang, Malaysia; 9 Department of Process and Food Engineering, Universiti Putra Malaysia, Serdang, Malaysia

**Keywords:** *Hydropuntia* sp., Polysaccharides, Seaweed, Biopolymer, Marine food

## Abstract

The red seaweed *Hydropuntia edulis* is found in Southeast Asia in the Indian and Pacific Oceans. Due to ease of cultivation and a greater concentration of sulfated polysaccharides, *H. edulis* is a superior source for agar production compared to other species. However, the pretreatments and extraction conditions strongly impact agar’s final gel strength and yield. Agar is made up of agarose, which is approximately 70%, and agaropectin, 30%, and has found a wide range of applications as a thickening, gelling, and stabilizing agent. This review highlighted agar extraction, its application in food as an ingredient, coating, and packaging. In-depth discussions about the interaction of agar with non-agar biopolymers have been made to diversify its utility in different avenues. Additionally, by considering the richness of bioactive derivatives of *H. edulis*, the applications in pharmaceuticals, agriculture, and aquaculture have been elaborated, followed by some limitations. Detailed investigation of *H. edulis* review aims to encourage increased cultivation of the species for producing agar and other bioactive compounds that support sustainable food, pharmaceutical, agriculture, and aquaculture industries.

## Introduction

The seaweed industry has attracted global attention, as it has the potential to support food security, especially by cultivating red seaweed. Since 2019, red seaweed production has increased to 18.3 million tons annually. The industry began in the 1950s and increased production from 2.2 to 35.8 million tons by 2019 (Cai et al., 2021). Red seaweed is abundant in marine coastal areas but uncommon in freshwaters. Among other red seaweed species, such as *Gelidiella* sp. and *Gelidium* sp., the *Hydropuntia* sp. is an important agar source worldwide (Vuai & Mpatani, 2019). *H. edulis* is a red seaweed found in Andaman Island, which is located in the Indian Ocean, the Pacific Ocean (China, Japan, and northeastern Australia), and Southeast Asia (Myanmar, Thailand, Vietnam, Malaysia, Indonesia, and the Philippines) (Assaw et al., 2018). *H. edulis* (2004) was previously known as *Rhodymenia cuneifolia* (1934) and *Gracilaria edulis* (1989), respectively (Bhushan et al., 2023).

**Figure 1:**
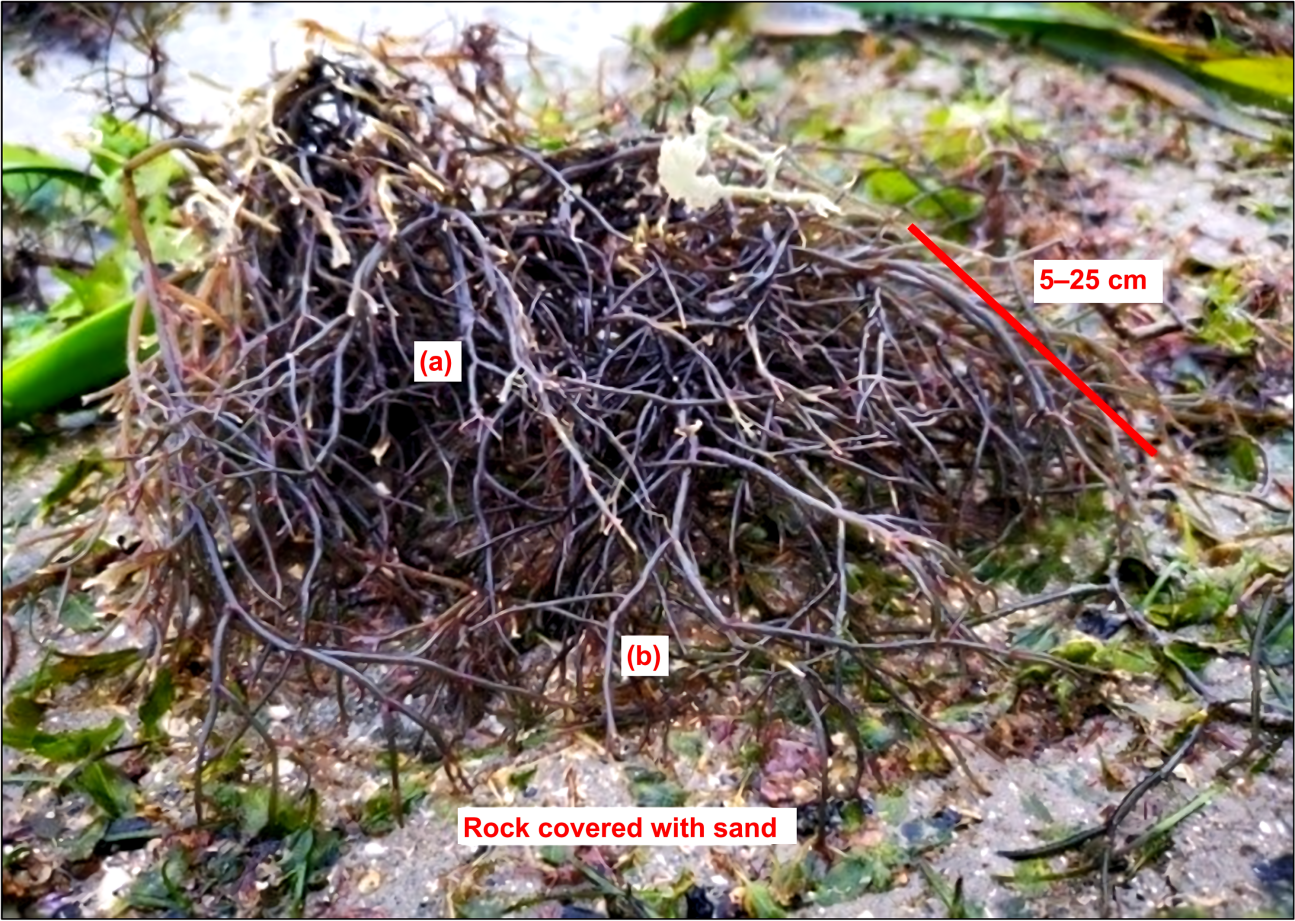
*Hydropuntia edulis*: (A) branches thallus; (B) thallus (with length of 5–25 cm). Source: Personal collection of Mohd Fakhrulddin Ismail.

*Hydropuntia* sp. can be grown alongside other aquaculture species. For example, *G. manilaensis* can be cultivated in abandoned shrimp ponds, where the seaweed removes the dissolved nutrients from the excessive shrimp feed, purifies the water, and produces biomass for agar extraction (Phang, 2006). In the mangroves of West Coast Peninsular Malaysia, *H. edulis* grows with *G. changii* and *G. salicornia*. Similarly, *H. edulis* and *G. crassa* grow well in the reservoir ponds used for salt production. The nets with seedlings are fixed with bamboo poles, and the plants are collected after three months of farming with a yield of 15–20 kg per net (Kyaw et al., 2009). Typically, *H. edulis* grows in clusters or bushy, with many branches arising from a base and anchored by a small discoid holdfast. The thallus of *H. edulis* is cylindrical, dark purplish red, succulent to slightly cartilaginous in texture, attaining 5–25 cm in length and 1–2 mm in diameter, and grows on rocks covered with sand in the lower intertidal zones of the open sea (Fig. 1) (Phang, Yeong & Lim, 2019).

Salinity, pH, and temperature are among the environmental variables have a major impact on the growth of *Hydropuntia* sp. Moreover, this seaweed can thrive in a wide range of salinities, from 10 to 40 PSU (Practical Salinity Unit), with the most efficient development occurring between 25 and 33 PSU. The optimal salinity range for thalli growth at 30 °C is between 20 and 30 PSU. It has a maximum daily growth rate of 2.58% at 30 °C and 25% under a 16:8 light/dark cycle (Kyaw et al., 2009). The response of seaweed to salinity varies according to the growing area, particularly for temperate species. As Chiaramonte, Faria & Plastino (2024) highlighted, *G. caudata* harvested from Brazilian coastal waters showed the highest growth rate of 12.6% at the optimum temperature of 26.1 °C.

Protein, minerals, and bioactive compounds are among the important macro- and micronutrients for human health that are abundant in *H. edulis*. It has been emphasized that *H. edulis* contains up to 25.29% protein, with almost all the essential and non-essential amino acids, making it a complete protein source (Rosemary et al., 2019). It also contains carbohydrates such as floridean starch (α-1,4-linked glucan), cellulose, xylan, and mannan. The water-soluble fiber fraction is formed by sulfur-containing galactans such as agar and carrageenan. Several studies have shown that *H. edulis* is a potential source of mineral supplements for human consumption. Similarly, the total lipids in *H. edulis* are 3.89 g/100 g. They are comprised of margaric acid (0.15 g/100 g), palmitic acid (0.63 g/100 g), oleic acid (1.05 g/100 g), stearic acid (0.93 g/100 g), linoleic acid (0.65 g/100 g), α-linoleic acid (0.14 g/100 g), stearidonic acid (0.22 g/100 g), and docosahexaenoic acid (0.12 g/100 g). Most seaweeds contain eicosapentaenoic acid (an ω-3) ranging between 2.4% and 10.7%, and *H. edulis* has the highest percentage of this fatty acid (Phang, 2006; Phang, Yeong & Lim, 2019; Rosemary et al., 2019). *H. edulis* is also packed with vitamins and could make an excellent nutrient-rich food source. Several vitamins have been discovered in *H. edulis*, including B1 (0.36 mg/g), B2 (1.54 mg/g), B3 (1.10 mg/g), B6 (4.77 mg/g), B9 (0.45 mg/g), C (13.41 mg/g), A (2.07 mg/g), E (1.49 mg/g), and vitamin precursors such as carotenoids (2.99 µg/g) (Rosemary et al., 2019).

Several bioactive compounds with anti-inflammatory, anticancerous, antidiabetic, and antioxidant activity are present in *H. edulis*, contributing to its health advantages. The presence of eugenol, nonane, hept-2-ene, 2,4,4,6-tetramethyl, undecane, 2-ethylhexyl isohexyl ester, hexatriacontane, sulfurous acid, phthalic acid, octacosane, 1,2-propanediol, and pentatriacontane has been reported in *H. edulis* (Arulkumar et al., 2018). Similarly, bioactive phenolic compounds such as gallic acid, gentistic acid, protocatechuic acid, hesperidin, rutin, p-hydroxybenzaldehyde, tyrosol, p-hydroxyphenyl acetic acid, and p-hydroxybenzoic acid are also found in this seaweed. Phloroglucinol is more potent than other polyphenols in terrestrial plants due to its potent antioxidant qualities and increased capacity to scavenge free radicals (Cai et al., 2021).

Archaeological evidence from Chile indicates that people first used red macroalgae during the Neolithic period, some 14,000 years ago. Individuals living in coastal areas have been gathering and harvesting certain types of seaweed for their own use. This approach has its roots in ancient civilizations such as Japan (13000 BC), China (2700 BC), Egypt (1550 BC), and India (300 BC), where aquatic plants were employed in traditional folk medicine to treat a range of ailments. In the Mediterranean region, red seaweed was utilized for medical and animal feed applications and as a dye during the ancient Greek and Roman periods. The red seaweed discovered in the Mediterranean has been utilized to combat parasitic worms, a practice originating in the pre-Christian era (Ali Ahmed et al., 2017). Only wild seaweed was available then, limiting its use as a food source until the Middle Ages. Many seaweed species have been used for more than six centuries in traditional medicine and the culinary arts in many parts of the world, particularly in East Asian nations like China, Japan, and Korea (Choudhary, Chauhan & Mishra, 2021).

*Hydropuntia* sp. has the highest economic value of any seaweed due to its agar content, which varies from 19% to 30% of the dry weight. With an estimated economic value of $2 billion USD, approximately 3.6 million tonnes of this fresh seaweed (10.5% of total production) were harvested in 2019, placing it third among all seaweed genera. *Hydropuntia sp.* alone accounts for 66–80% of global agar production (Cai et al., 2021). Compared to agar made from other seaweed species, this agar is more stable and has a higher melting temperature (65 °C) (De Alwis & Wijesekara, 2022). Given its robustness and higher agar yield, *H. edulis* is far more practical to cultivate for agar production than other seaweed species (Kuragodage, Cumaranatunga & Deepananda, 2022).

According to the published literature trend from 2010 to 2025 (Table 1), a plethora of research has been found regarding the seaweeds and only a few review articles focus on the *H. edulis*. Notably, these published studies focus on the *H. edulis* cultivation, phylogenetic evaluation, and extraction of novel bioactive compounds of pharmacological significance (*i.e.,* antimicrobial, immunomodulating, antiproliferative, antidiabetic, and anti-obesity). However, no specific review has been published scoping the comprehensive nutritional value of *H. edulis* such as its agar extraction, bioactivities, and utilization in food and non-food sectors. Therefore, this review focuses on the potential of *H. edulis* and its valuable applications. Especially, as a new source of functional ingredients for multifaceted uses in food applications. Besides, the non-food applications, such as medicinal, agricultural, and aquacultural, of the *H. edulis* derivatives have also been highlighted, which would benefit industrial production and contribute to sustainable aquaculture (Narayanan & Sudhakar, 2025). Other than that, the blending properties of the agar and other biopolymers are also the gist of the review, which has not been discussed in other literatures.

**Table 1:** The literature (research/review articles) publishing trend for *H. edulis* between 2010 and 2025.

**Objective**	**Key sections/findings**	**References**
***Review articles***		
The pharmacological activity and bioactivities of marine macroalgae in Indian coast including *H. edulis*	– Elaborated the potential bioactivities and pharmacological activities of *H. edulis*. – *H. edulis* positively affected the antidiabetic activity of STZ-induced diabetic rats. – The main bioactive phytochemical of *H. edulis* was phytosterol. – Other seaweed species (green and brown) were also discussed.	Sharma, Koneri & Jha (2019)
Reviewing biological activity marine red algae compounds towards skin whitening including *H. edulis*	– *H. edulis* has higher antioxidative activity than *Hynae valentiae.* – Butanolic extract of *H. edulis* showed the highest ABTS antioxidant activity and polyphenol content. – Red algae have the potential to be used as whitening agent due to its antioxidant properties like ascorbic acid, BHT and BHA.	Sheba, Baharulnizam & Rajabalaya (2022)
Elaborating the biology of *H. edulis* and its farming industries as well as its application in various industries	– *H. edulis’* most common farming methods are the raft and tube net cultivation method, which is propagated using seed material and carpospores, a spore-based planting material. – Economically important for its food grade agar and used as plant bio-stimulant.	Bhushan et al. (2023)
Highlighting the biological activity potential of marine macroalgae including *H. edulis*	– Polysaccharides of marine macroalgae are essential nutritional components. – They are used for producing various products such as hydrogels, food stabilizers, agar, and others. – *H. edulis* has been reported to possess anti-hyperglycemic and antidiabetic activity.	Cadar et al. (2025)
***Research studies***		
Protein isolated from *H. edulis*	– *H. edulis* protein isolation and characterization was made. – The aqueous and methanol extracts yielded approximately 12 g/kg protein. – SDS-PAGE yielded four well-defined bands at 31.4, 69.5, 92.7 kDa in both the extracts. – The extracts showed highest antioxidant activity (80–95%) *via* DPPH assay.	Boobathy et al. (2010)
Green synthesis of silver nano particles from *H. edulis*	– Silver nanoparticles were obtained from *H. edulis via* green synthesis. – Nanoparticles with the size range of 12.5–100 nm were obtained.	Murugesan, Elumalai & Dhamotharan (2011)
*H. edulis* in improving the water quality of a shrimp pond	– The findings revealed that *H. edulis* potentially reduced the excessive nutrient residues of the brackish water that led to eutrophication. – It efficiently lowered NO_3_^−^ (42%), NH${}_{4}^{+}$, (60%), and PO_4_^3−^ (57%) in the pond water. – *H. edulis* possessed the most UV-absorbing compounds. – *H. edulis* efficient in ammonia and phosphate reduction.	Baloo, Kikuchi & Azman (2011), Lavania et al. (2012), Tanaka et al. (2020) and Sarkar et al. (2021)
Antifungal activity of *H. edulis*	−*H. edulis* has the antifungal compounds evaluated by thin layer chromatography and gas chromatography/mass spectrometry (GCMS) especially terpenes.	Peres et al. (2012)
Boosting immune response of *Macrobrachium rosenbergii* immersed in hot-waterextract of *H. edulis*	– Hot water extract of *H. edulis* enhanced the immune response of *Macrobrachium rosenbergii* (shrimp). – Increased total haemocyte count (THC), greater phenoloxidase (PO) activity, and percentage survival against *V. alginolyticus* were observed after the immersion in tanks with 0.1g/L extract.	Maningas et al. (2013)
*H. edulis* extract inhibits tumour in Ehrlich Ascites tumour cells *in vivo*	– Ethanol extract of *H. edulis* increased the lifespan of Ehrlich ascites tumor (EAT)-bearing mice and inhibited tumor growth. – Daily dose of extract up to 300 mg/kg for 35 days did not show toxicity.	Patra & Muthuraman (2013)
Production of ethanol-from-cellulose from cheap sources seaweed *H. edulis*	– Ethanol was produced from *H. edulis* by fermentation method using *Saccharomyces cerevisiae* and *Aspergillus niger.* – The presence of ethanol was detected using GCMS.	Amanullah et al. (2013)
Synthesis of metallicsilver and zinc oxide nanoparticles from *H. edulis* and the anticancer activity against human PC3 cell lines	– Microwave-mediated protocol was applied for extracellular synthesis of metallic silver (Ag) and zinc oxide (ZnO) nanoparticles. – The inhibitory concentration values were found to be 39.60, 28.55, 53.99 μg/mL and 68.49, 88.05, 71.98 μg/mL against PC3 cells and Vero cells.	Priyadharshini et al. (2014)
*H. edulis* as antimicrobial agent	– Methanolic extract of *H. edulis* showed greater inhibitory activity than other solvents (acetone, petroleum ether, hexane and ethanol). – Inhibition was excellent for *Streptococcus pyogenes* followed by *Bacillus subtilis*, *Staphylococcus aureus*, *Streptococcus epidermis* and *Bacillus cereus*. – GCMS was used to analyze the bioactive compounds in *H. edulis*. – Molecular docking (Autodock 4.2.6) was used to visualize the drug properties to dock against Aerolysin, the virulent bacterial enzyme. – Eugenol has the best docking score −4.42 Kcal/mol, followed by 2 Heptane, 2,4,4,6 tetramethyl and 1,2-Propanediol with docking score of −3.89 Kcal/mol and 2.77 Kcal/mol, respectively.	Kolanijinathan & Saranraj (2014), Biswal & Pazhamalai (2019) and Asghar et al. (2021)
Evaluation of physicochemical properties, proximate and nutritional composition of *H. edulis* harvested from Palk Bay	– The red seaweed was consisting of carbohydrate (101.61 mg/g DW), dietary fiber (8.9% DW), lipid content (8.3 mg/g DW), and crude protein (6.68e mg/g DW). – Fatty acids such as linolenic acid (2.56%), palmitic acid (2.06%), and oleic acid (1.98%) were also detected. – Chlorophyl A and B, proline, vitamins (A, C, E) and essential amino acids were also found. – The total amino acids were 76.60 mg/g seaweed.	Sakthivel & Devi (2015); Gunathilaka et al. (2019) and Rosemary et al. (2019)
Antiproliferative potential of *H. edulis* extract	– Different solvent extracts (ethyl acetate, petroleum ether, dichloromethane, benzene, chloroform, methanol and water) were tested for its antiproliferative potential against A549 cancer cells. – Ethyl acetate extract showed a significant growth inhibition at 48 h with an IC_50_ of 24.5 μ g mL^−1^.	Sakthivel et al. (2016)
*H. edulis* as prebiotic edible dietary fiber	– *H. edulis* possess handsome amount of total dietary fiber (63.1%). – It has Na (423.3 mg 100 g^−1^), P (282.5 mg 100 g^−1^), Ca (223.3 mg 100 g^−1^) and Fe (65.2 mg 100 g^−1^). – *H. edulis* had higher vitamin D_2_ (2.590 mg 100 g^−1^), vitamin E (1.017 mg 100 g^−1^) and vitamin K_1_ (0.714 mg 100 g^−1^).	Debbarma et al. (2016)
COI-5P Gene sequence variation in Philippine *H. edulis* and farming as an eco-engineering strategy	– Phylogenetic and haplotype analyses were performed based on mtDNA COI-5P gene sequences. – A total of 14 haplotypes were recovered, of which seven were newly detected. – *H. edulis* can be successfully grown in the mid- and upper-intertidal zones on seawalls by using planters with water-retaining features.	Ferrer, Gomez & Dumilag (2020); Heery et al. (2020)
Identification of new milk-clotting proteases from *H. edulis*	−Two protease bands with a molecular weight of 44 and 108 kDa were identified.	Arbita et al. (2022)
Potential of water-soluble poultry manure promoting growth and enhance the biomass production of *H. edulis*	– Poultry manure was prepared in aqueous extract and mixed with seawater. – This manure demonstrated higher growth rate than seawater alone.	Narayanan & Sudhakar (2025)
Evaluate multitarget bioactive compounds in *H. edulis* with antidiabetic and anti-obesity activities	– The extract inhibited α-amylase, α-glucosidase, and DPP-4 in a dose-dependent manner. – The extract lowered fasting blood glucose, reduced weight gain, improved lipid profiles, and modulated key proteins involved in insulin and lipid signalling.	Savitri et al. (2025)

**Notes.**

*Hydropuntia edulis (H. edulis)* is the new taxonomic name for *Gracilaria edulis (G. edulis)* but still used interchangeably in literature.

## Search Methodology

**Figure 2:**
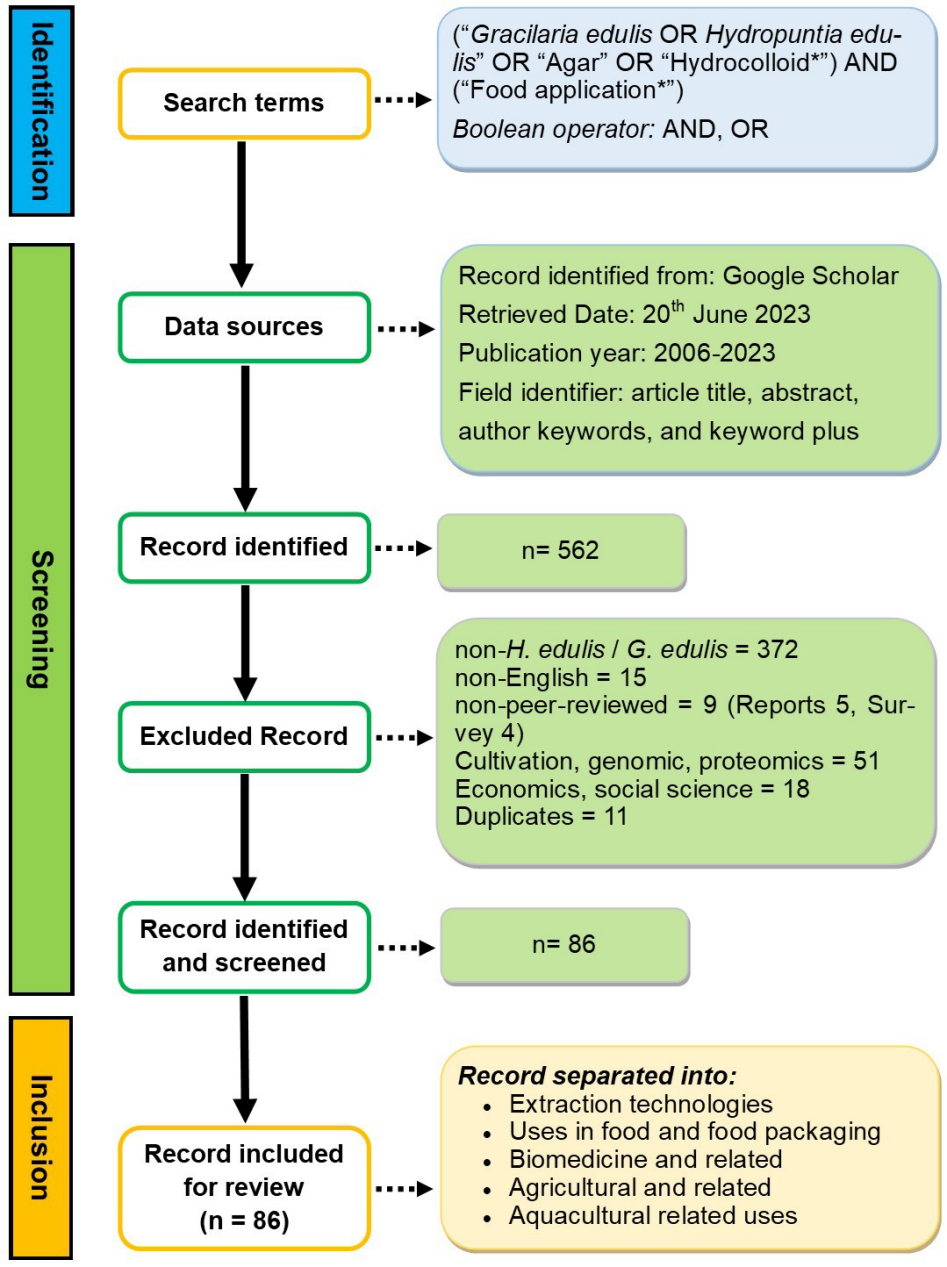
A schematic overview of the survey methodology, depicting the various stages, identification of the literature (search terms), screening (data sources, record identified, excluded record, record identified, and screened), and inclusion (record included for review).

Figure 2 shows the schematic overview of the survey methodology, depicting the various stages, from identification of the literature (search terms), screening (data sources, record identified, excluded record, record identified, and screen), and inclusion (record included for review, *n* = 86). The research documents utilized for this review were searched on 18th October 2025. The search was refined using the Boolean operators “OR” and “AND”, combined with the phrases *Gracilaria edulis, Hydropuntia edulis*, and agar. The search query employed was (“*Gracilaria edulis” OR “Hydropuntia edulis*” OR “Agar” OR “Hydrocolloid*”) and (“Food application*”) within the accessible literature on Google Scholar, utilizing the filter for “article title, abstract, author keywords, and keyword plus”. Researchers conducted a thorough literature review of the chosen search query (keywords) utilized in the title, abstract, or keywords when finding publications on seaweeds’ food applications. Employing quotation marks (””) will yield only the precise phrase, whereas utilizing an asterisk (*) will produce both singular and plural variations of keywords. The Google Scholar database is perpetually updated, albeit with modest adjustments. Consequently, the obtained number of publications may exhibit minor variations, although employing the identical retrieval method on a different date. A time span (2006–2025) was designated to examine the recent trends in the research field for *H. edulis*. The Google Scholar database returned 562 documents. The criteria of inclusion focused on peer-reviewed studies from 2006 to 2025 that investigated “*H. edulis*” red seaweed, specifically its extraction, bioactivity, and applications, while excluding studies on “non-*H. edulis*”, non-English publications, and non-peer-reviewed sources that lacked scientific rigor. Only 86 studies were identified that met the screening criteria. The literature was analyzed by organizing studies into themes and integrating findings regarding methodology, attributes, and applications. Every pertinent detail from the titles was included, emphasizing patterns and contributions to the review’s goal. Five research topics were formulated to examine these themes, concentrating on extraction technologies, food and food packaging uses, biomedicine, and agricultural and aquacultural sustainability. The aim was to consolidate existing knowledge on the properties and applications of *H. edulis*, assess its potential in food, biomedical, and agro-environmental domains, and delineate future research avenues.

## Discussion of literature synthesis

### Agar from *H. edulis*

The genus *Hydropuntia* is a source of agar, which is a mixture of agarose and agaropectin. Agarose is a natural substance that forms strong gels and is made of repeating units called agarobiose, which are made up of two sugars: D-galactose and 3,6-anhydro-L-galactopyranose, connected by specific types of chemical bonds. Agaropectin is a sulfated polysaccharide with a low gel strength, which is made up of D-glucuronic acid and pyruvic acid. It is a non-uniform molecule with a structure similar to agarose, yet branched and sulfated (Arham et al., 2016; Roy & Rhim, 2022). *Hydropuntia sp.* is more valued than *Gelidium sp.* based on its higher agar yield. Consequently, the majority of agar is currently supplied by *Hydropuntia,* which includes *H. edulis, H. fergusonii, H. millardetii, H. corticata, and H. salicornia.* Since 2019, most agar has come from *Hydropuntia sp.* cultivation in China (95.63%), with a relatively minor quantity from wild collection in Chile (Cai et al., 2021).

Agar does not raise the net calories of food as it possesses over 94% soluble fiber (Sousa et al., 2010). Of the total agar consumption, about 80% of agar is utilized in food applications, including water gels, fermented items, dairy goods, fish products, canned meats, and as a clarifying agent in juice, vinegar, and wine. However, the pharmaceutical and biotechnology sectors hold the remaining 20% (Khalil et al., 2018). Numerous aspects impact the economic worth and quality of the seaweeds’ agar, such as season, light intensity, salinity, temperature, water depth, epiphytes and epibionts, physiological condition, nutritional status, extraction process, and storage conditions (Kyaw et al., 2009). Agar is soluble in boiling water, with a clear aqueous solution forming a gel between 32−43 °C. It can form reversible and strong gels with a high melting point simply by cooling the hot aqueous solution (Khalil et al., 2018). Agar’s strength, gelling and melting temperatures, thickness, syneresis, and chemical characteristics, particularly the amount of sulfate and 3,6-anyhydrogalactose, all affect its quality. The primary indicator of agar quality is the gel strength, which can be measured using 1.5% w/v agar gel according to the Nikkansui method (Siow et al., 2012). Various hydroxyl groups in galactose can be substituted by sulfuric acid, methyl, and pyruvic acid, and the positions affect the gel strength of agar. Sousa et al. (2010) reported that the sulfate content in the extracted agar from *Hydropuntia sp.* strongly impacts the gel properties.

The solution-gel transition of agar undergoes hysteresis around 40–60 °C. The hysteresis is a phenomenon in which, when agar is mixed with water, it solidifies into a gel at 32–42 °C and will be melted when heated above 60 °C. The thermal hysteresis decreases due to the high degree of agar sulfation. Generally, a higher level of sulfation prevents large helices from sticking together and disrupts the hydrogen bonds between molecules. A superior quality of agar normally has a wide range of hysteresis and few side chain substitutions. The changes in structure that can happen after sulfation influence the physical and flow properties of the agar (Balqis et al., 2017). The viscosity of agar solution is also an important indicator of its quality and is directly proportional to the molecular weight of the agar. A low substitution of a charged group in agar results in a low-viscosity agar (Freile-Pelegrin, Azamar & Robledo, 2011). The phenomenon of losing water over time in agar hydrogel is known as gel syneresis, which is largely affected by the aggregation of double helices, which directly affects the polymer network contraction and leads to a reduction of interstitial space available for water holding (Kyaw et al., 2009). As for other factors, such as agar concentration and storage time, the sulfate contents have a stronger impact on gel syneresis.

The agar extraction from *Hydropuntia* sp. is generally performed by leaching the dried seaweed in boiling water, filtering the extract, and separating the agar by freeze-and-thaw techniques. However, the freeze-and-thaw method is relatively expensive due to the refrigeration cost. In a study by Priyadharshini et al. (2014), the seaweeds were soaked in purified water and autoclaved at 121 °C for 15 min to obtain the agar extract. The liquid extract was further purified by double filtration using a steel gauge and cellulose filter paper, and then freeze-thawed prior to dehydrating and drying at almost 50 °C. Similarly, a 7% w/v seaweed aqueous suspension was autoclaved before agar extraction and provided a better agar yield than the water bath extraction method (Kyaw et al., 2009). This disparity in agar yield could be attributed to the better extraction power of the autoclave, where high pressure and heat are employed, which better disrupts the cell wall and releases the polysaccharides, contrary to just heat application in a water bath extraction. However, the adoption of longer autoclaving (60 min) of seaweed suspension in distilled water (1:3 solid to liquid ratio) provided agar extract with better clarity without having any solid particles that were freeze-thawed and dried to obtain powdered agar (Assaw et al., 2018).

The agar extraction method has also been optimized by applying some pretreatments, such as alkali treatment, to increase the agar gel strength for commercial use. However, the results obtained by Sousa et al. (2010) confirmed that increasing the alkali concentration reduced the agar yield. The results indicated that the lower concentration of alkali (1%) resulted in a greater yield of agar compared to the concentrated (6%) alkali treatment. The decrease in agar yield due to higher alkali concentration might be related to the decomposition of polysaccharides and the diffusion into the solution during the alkali treatment. Alkali converts L-galactose-6-sulfate to 3,6-anyhydro-L-galactose in the agar structure and thus increases the agar yield of *Hydropuntia* sp. to compensate for the lacking quantities of agar from the *Gelidium* sp. (Sousa et al., 2010). However, Lähteenmäki-Uutela et al. (2021) employed a water bath extraction method at 85 °C for 120 min and a lower alkali concentration (2.5%) in the pretreatment. After neutralizing the seaweed, the extract was heat-treated for 120 min at 120–125 °C and then freeze-thawed and dried for 24 h at 60 °C.

Agar from seaweed was extracted using the cold method and microwave-assisted extraction (MAE) by Kyaw et al. (2009). MAE was carried out at a higher temperature to speed up the movement of agar from the seaweed matrix into the solvent. After being pretreated with 6% NaOH for 3.5 h at 85 °C, the seaweed was neutralized for 60 min with 0.5% acetic acid and then washed with distilled water. Following extraction, the solution was clarified by filtration using a cloth and recovered by a freeze-thaw procedure. The agar obtained was washed with 96% ethanol, then oven-dried at 60 °C. The yield of agar from the seaweed was about 6–71% of total dry weight (Lee et al., 2017). However, in one study, agar was extracted using methanol, an organic solvent, with a solid-to-solvent ratio of 1:10. The extract was concentrated using a vacuum-pressured rotary evaporator and finally dried in an oven to obtain dried agar (Assaw et al., 2018). The amount of agar produced from *H. edulis* varied between 19.9% and 28.4%, and it has a gel strength of 78–167 g cm^−2^ (Priyadharshini et al., 2014; Sakthivel & Devi, 2015; Lähteenmäki-Uutela et al., 2021).

Thus, the alkaline pretreatment up to a certain concentration could improve agar yield; however, in both temperate and tropical regions, the correlation between biomass and agar production is dependent on the species of the seaweeds (Sakthivel & Devi, 2015). Among the reported methods, alkali treatment and autoclave techniques provide optimal yields and gel strength, establishing them as industry benchmarks; nonetheless, they are less environmentally sustainable. These two methods are more adaptable across species and are scalable. On the other hand, although MAE and freeze-thaw methods are more ecologically friendly, they may compromise yield, require optimization, and present financial challenges. Regarding sustainability, the MAE and freeze-thaw procedures are more congruent with green technology trends, whereas alkali and methanol treatments pose environmental concerns due to chemical waste.

### Molecular interaction of seaweed agar blended with different types of polysaccharides

The primary structure of polysaccharides such as agar is mainly influenced by the features of sugar constituents (*e.g.*, molecular weights, degree of branching, presence of functional groups, and glycosidic linkages), which then contribute to biological activities and physicochemical properties of the polysaccharides (Popović et al., 2018). Moreover, agar blends are normally developed and employed for different applications to tailor their properties. For instance, agar, due to its gelling capability, reversibility, and melting, is employed to develop agar films which offer high heat-sealability, biodegradability, and transparency (Armisen & Gaiatas, 2009). However, the agar-based film is relatively fragile compared to plastic packaging, showing higher moisture permeability and poor elasticity. Thus, integrating agar with other substances such as biopolymers, plasticizers, hydrophobic materials, antimicrobial agents, and nanoparticles may improve the functional qualities of agar films (Mostafavi & Zaeim, 2020).

In effort to improve the agar film properties, polymer blending has been employed by mixing the agar with other biopolymers such as arabinoxylans, starch, lignin, *κ*-carrageenan, and locust bean gum (Khalil et al., 2018; Polat, Duman & Tunc, 2020; Panggabean et al., 2022). Naik, Wang & Selomulya (2022) blended agar from *Gracilaria secundata* with amaranth protein in powder form. It shows that an alternative spray drying method can be used instead of freeze-drying or casting it into a film. Initially, the solution was treated with ultrasound pretreatment that could enhance the bonding interaction of the two biopolymers, and finally spray-dried by maintaining the inlet and outlet temperatures of 175 °C and 80 °C, respectively. The developed powder was homogenous and stable, which could be attributed to the possible intermolecular interactions (hydrogen bonding and van der Waals forces) between the polar group of amino acids in protein and the hydroxyl group of agar (Naik, Wang & Selomulya, 2022). Employing spray drying instead of traditional freeze-drying or casting offers a scalable and cost-effective method for producing biodegradable powder-form materials, suitable for applications like food packaging or coatings.

Wolska, Setkowicz & Maliszewska (2020) developed an active wound dressing by blending various ratios of agar and chitosan. The chitosan solution was prepared by dissolving chitosan in 2% acetic acid that contained 0.4% NaCl (sodium chloride). The agar solution was prepared by dissolving 2% agar and heating it in a microwave oven. Then, 50 mL of 2% chitosan solution was poured into a flask and placed in the water bath (70 °C) on top of the magnetic stirrer. Once the temperature was 90 °C, agar solution was added and continued for 15 min of stirring. Lastly, the mixture of chitosan and agar solution was cast onto a petri dish and allowed to solidify before evaluation. The results showed that the best chitosan to agar ratio was 2:1, where the developed film was flexible, transparent, and had satisfactory coherence. Because chitosan is cationic in acidic environments, the hydroxyl group in agar and the amino and hydroxyl groups in chitosan probably encouraged hydrogen bonding and electrostatic interactions. Strength, flexibility, and bioactivity can all be improved by customizing the film’s properties for particular uses by varying the chitosan-to-agar ratio.

Another cost-effective method of producing biodegradable films is blending starches with seaweed agar due to the inexpensive nature of the starch. Adding agar to the maize starch biopolymer considerably increased the tensile strength and flexibility of the films (Westlake et al., 2022). The inhibition of starch chain connections and rearrangements seen by Fourier transform infrared spectroscopy (FTIR) analysis suggests that the galactan chains in agar may have disrupted the hydrogen-bonded network of starch molecules. This change was probably brought about by competing hydrogen bonds between the starch and agar hydroxyl groups. Furthermore, the contact angle measurements demonstrated that the starch-agar blend film became less hydrophilic. Thus, the polymers in the blend, their ratio and concentration, and the chemical nature could upgrade the agar functional properties for tailored applications (Freile-Pelegrin, Azamar & Robledo, 2011). Table 2 summarizes the findings related to blends of agar, alginate, and carrageenan blend films with other polysaccharides based on the literature.

**Table 2:** Blending of marine polysaccharides including agar with other biopolymers.

**Biopolymer blends/composites**	**Film properties**	**Application**	**References**
*Agar*			
Agar/chitosan (1:2 ratio)	– Good film flexibility – Increased transparency – High moisture sorption (>160 moisture sorption rate %) after 1h	Hydrogel film as wound dressings	Wolska, Setkowicz & Maliszewska (2020)
Agar/k-carrageenan/montmorillonite nanocomposite hydrogels	– Hydrogel films controlled the release of antibiotic chloramphenicol (CLP) (91.04–72.84%) – Controlled release of analgesic lidocaine hydrochloride (LDC) (88.8–73.4%)	Controlling of the released of drugs in the hydrogels	Polat, Duman & Tunc (2020)
Sugar palm starch/agar	– Increased tensile strength up to 56% (>20 MPa) – Improved Modulus by 78.6% (>3500 MPa)	Thermoplastic films	Jumaidin et al. (2017)
Agar/soy protein isolate (SPI)	– Flexible film – Transparent film – Presence of hydrogen bonding and hydrophobic groups in the composite films (FTIR-Fourier Transform Infrared)	Potential biodegradable and renewable materials as alternative to plastic packaging	Guerrero et al. (2014)
Starch/agar composite films blended with silver nanoparticles (AgNPs, derived from Enoki mushroom water extract)	– Improved the hydrophobicity – Improved water vapor barrier of composite films from 0.75 to 0.62 (×10^−9^ gm/m^2^.Pa.s) – Antibacterial film against foodborne pathogenic bacteria (*E. coli*; stopped growing in 6 h, and *L. monocytogenes*; stopped growing in 12 h)	Antibacterial film	Roy & Rhim (2022)
Thermoplastic sago starch/agar	– Improved the tensile, flexural and impact properties of the films – 10 wt% agar showed the highest impact strength – Good interfacial adhesion – Maximum tensile strength of 8.61 MPa – Young’s Modulus of 995 MPa – Increased thermal stability with the presence of agar	Thermoplastic sago starch film	Roy & Rhim (2022)
Agar/nano-bacterial cellulose (BC)	– Low concentration of BC in the films (3–5%) showed a good dispersion of BC – FTIR and rheology suggest hydrogen bondings between agar and bacterial cellulose (BC) – Addition of BC improved the thermal stability and crystallinity of the films – High concentration of BC (10%) reduced the water solubility, moisture content and water permeability of films	Potential agar-based packaging films	Wang et al. (2018)
Agar/paper-mulberry pulp nanocellulose (CNC)	– Increased tensile strength (25%) and modulus (40%) with 5 wt% CNC film – Decreased water vapor permeability of composite film with 3 wt% CNC by 25%	Agar-based bionanocomposite films	Reddy & Rhim (2014)
*Alginate*			
Ternary blend film of sodium alginate (SA)/carboxymethyl cellulose (CMC)/potatostarch (PS)	– Reduced the UV transmittance to 1.89% – Tensile strength increased 31.95 MPa – Elongation was 13.78% – Moisture content of 16.21%	Enhance the post-harvest shelf life of black and green grapes by up to 16 days	Ramakrishnan et al. (2022)
Betalain-based from red prickly pear covalently linked into cellulose/alginate blend films	– pH below 10 shows purple color and above 10 give yellow color – Maximum swelling in water was 455 wt% – Young’s modulus of 2,086 MPa – Tensile strength of 68 MPa – Strain at yield was 7%	pH sensitive smart packaging films	Halloub et al. (2023)
Sodium alginate/gelatin/ poly(vinylalcohol) blend films incorporated with catechin and tannins	– Improved water insolubility – Enhanced barrier performance – Increased antioxidant activity – Higher tensile strength with 2% tannins compared to catechin modified films	Packaging films	Liu et al. (2023)
Starch/sodiumAlginate/montmorillonite films	– Improved barrier properties – Improved optical properties – Increased thermal stability – Enhanced mechanical properties – Degrade in soil after 22 days	Antibacterial films	Zhang, Li & Jiang (2020)
*Carrageenan*			
Arrowhead (*Sagittaria sagittifolia*) starch/ *κ*-carrageenan and blackchokeberry (Aronia *melanocarpa*) extract	– Decreased light transmittance – Increased thickness and elongation-at-break	Antioxidant and pH-sensitive films to chicken wings	Wang et al. (2018)
*κ*-carrageenan/carboxylated cellulose nanofibril/phytic acid	– Improved elongation-at-break (41%) – Improved water vapor permeability (3.46 g m^−1^ Pa^−1^ s ^−1^×10^−8^) – Improved oxygen permeability (1.50 cm^3^ mm^−2^ Pa^−1^ day^−1^×10^−12^) – Good thermal stability, limiting oxygen index and actual flame-burning behavior	Flame-retardant and antibacterial films for pork preservation	Ning et al. (2023)
Starch/*κ*-carrageenan/*Oxalis triangularis* extract film	– Increased thickness – Increased water vapor permeability – Increased light barrier – Improved tensile strength and elongation-at- break – Improved the pH- and ammonia-sensitive properties	Antioxidant and ammonia-sensitive smart packaging films as indicator of beef meat spoilage	Jiang et al. (2023)

### Potential application of *H. edulis*

#### *H. edulis* in food applications

##### As a food ingredient.

*H. edulis* is commonly used as a source of food ingredients due to the greater amounts of proteins and vitamins. Nielsen, Rustad & Holdt (2021) reported the highest vitamin C with a value greater than 3.0 mg/g in *H. edulis*, which is higher than in other seaweeds in the study. Another work revealed that the red seaweed provides significant amounts of iron (14.8–72 mg/100 g), iodine (38.8–72.2 mg/100 g), and calcium (410–870 mg/100 g) (Thambi & Chakraborty, 2023). The extraction methods, geolocation, and cultivation conditions could be the reason for these significant variations in mineral contents among different studies. *Hydropuntia* sp. has been used in manufacturing foods such as noodles using freeze-dried flour at different concentrations, *i.e.,* 0, 1, 3, 5, and 7% w/v. The noodles with 3% seaweed depicted a significantly greater dietary fiber content, whereas consumers showed moderate sensory acceptability (Keyimu, 2023). Improving seaweed agar-based noodles’ sensory qualities requires a multifaceted approach that includes color enhancement, texture improvement, and taste masking. Using techniques like flour amalgamation, natural taste enhancers, and processing modifications could also significantly increase consumer appeal. However, subsequent research should concentrate on optimizing seaweed content, investigating component molecular interactions, and performing comprehensive sensory assessments with varied trained panels to ensure sensory attractiveness and marketability of seaweed-based products to a wider audience.

##### As a prebiotic.

Debbarma et al. (2016) reported that *H. edulis* has the highest total dietary fiber (63.17%) as compared to other seaweeds, such as *Sargassum* sp. (58.25%) and *Ulva lactuca* (53.63%). A diet containing agar has been developed to assist patients with impaired blood glucose and obesity. The results indicated that the agar addition resulted in notable weight loss for consumers due to reduced calorie intake. It was reported that seaweed polysaccharides are indigestible carbohydrates that act as superior prebiotic and a source of energy for gut microbiota (probiotic). Gut microbiota plays a vital role in host metabolism and contributes significantly to the development and regulation of the immune system. Studies have reported approximately 10^14^ bacterial cells in the adult human microbiome (Mostafavi & Zaeim, 2020; Charoensiddhi et al., 2022). Due to the backbone structure of seaweed polysaccharides, it is difficult for digestive enzymes to break down these carbohydrates. Therefore, the gut bacteria ferment the carbohydrates into short-chain fatty acids (SCFA), which in turn promote the growth of the good bacteria and contribute to improved immune systems as well as digestion and absorption mechanisms in the host body (Charoensiddhi et al., 2022).

According to Lopez-Santamarina et al. (2020), seaweed-derived polysaccharides and oligosaccharides regulate intestinal metabolism by preventing the invasion and adhesion of pathogenic bacteria and treating the inflammation caused by bowel disease. In contrast, a lack of polysaccharides in the colon will allow the gut microbiota to consume amino acids and proteins as metabolic energy sources. This proteolytic fermentation produces some metabolites, including nitrogenous products such as ammonia, amines, and carcinogens. Many studies have indicated that seaweed polysaccharides are potential anti-inflammatory, anticancer, antioxidant, and anti-tumor agents (Lopez-Santamarina et al., 2020; Gomez-Zavaglia et al., 2019; Okolie et al., 2017; Rosa et al., 2019).

##### In food packaging.

*H. edulis*, as a potential source of agar, could be a beneficial material for the formulation of biodegradable, active, and intelligent packaging. Active packaging has functional properties such as antioxidant, antimicrobial, and a good gas barrier (Westlake et al., 2022). While, intelligent food packaging refers to a system that can monitor the conditions of packaged food during storage and transportation (Roy & Rhim, 2022). Several natural substances, like chitosan, starch, k-carrageenan, and locust bean gum, have been blended with agar to develop packaging films. Starch is the most widely studied material incorporated into agar due to its lower cost (Mostafavi & Zaeim, 2020).

Roy & Rhim (2022) have developed agar-based composite films blended with melanin nanoparticles as a potential biodegradable biopolymer packaging. Using centrifugation, melanin nanoparticles (MNP) were used as functional fillers extracted from sepia ink. Incorporating MNP into agar composite films improved the antioxidant activity and enhanced agar composite films’ mechanical, water vapor barrier, and hydrophobicity. The agar composite films’ tensile strength, flexibility, and stiffness were correlated with the addition of MNP. Adding 0.5−1.0% of MNP improved the tensile strength of films (36.1–46.7 MPa) compared to agar-only films (34.8 MPa). Similarly, elongation-at-break (EB) (12.2–16.1%) and Young’s modulus (EM) (1.2−2.1 GPa) were also higher than the control agar film. Furthermore, adding 2% MNP to the agar films raised its antioxidant activity from 7.4% to 47.1%.

Blending chitosan into the agar films strongly enhanced the films’ water vapor transmission rate, EB, and tensile strength. The addition of agar into chitosan film has been shown to increase the tensile strength of composite films (2.72 to 5.31 MPa) as agar concentration increased up to 40%. More flexible films were obtained when agar concentration was increased, as increased EB (2.5 to >4%) was noticed for composite films. Contrarily, the water vapor transmission rate (WVTR) increased from 800 to 1,200 g/m^2^-d as agar concentration increased in the composite films. WVTR in packaging plays an important role in preventing food deterioration by hindering moisture migration (Wolska, Setkowicz & Maliszewska, 2020).

#### In pharmaceutical industry

Approximately 70% of anticancer chemotherapeutic drugs nowadays are derived from natural resources, including marine flora such as seaweeds (Patra & Muthuraman, 2013; Bhushan et al., 2023). The secondary metabolite contents in marine products possess a great anticancer potency. Arc-C (Cytarabine), an antileukemic drug, and trabectedin, an agent for treating soft tissue sarcoma, is derived from marine sources. The ethanolic extract of *H. edulis* induced apoptosis and suppressed the tumor in Ehrlich ascites tumor cells *in vivo* and *in vitro* (Priyadharshini et al., 2014). Kasanah et al. (2019) reported that cholest-8-en-3-ol, eicosanoic acid, 13-octadecenoic acid, pentadecanoic acid, and 10-octadecenoic acid were the compounds from the active fraction of *H. edulis*, detected using gas chromatography-mass spectrometry, that are bioactive and have antibacterial properties. Sakthivel & Devi (2015) observed that *H. edulis* possesses higher fatty acid content, both saturated and unsaturated (palmitic acid, stearic acid, margaric acid, linolenic acid, and alpha-linolenic acid), compared to *H. acerosa*. Fatty acids augment the antibacterial effect of seaweed, as they help perforate the cell walls and rupture the membrane of bacteria, causing the cell to shrink and leading to cell death (Cadar et al., 2025).

As the seaweed extracts contain concentrated active secondary metabolites, their purified constituents have anticoagulant, antioxidant, antiviral, anti-inflammatory, and anticancer properties (Pérez, Falqué & Domínguez, 2016). Kumar & Fotedar (2009) reported that the seaweed extracts can reduce human oxidative stress and blood glucose. These bioactive agents provide substantial protection against pathogenic microorganisms. Antimicrobial activity assays conducted by Kolanijinathan & Saranraj (2014) proved that the extracts of *H. edulis* showed a great inhibition zone against Gram-positive bacteria such as *Streptococcus* sp.*, Bacillus subtilis,* and *Staphylococcus aureus*. In addition, the methanolic extract of *H. edulis* has shown a maximum inhibitory zone against fungi such as *Aspergillus* sp. (17–18 mm), *Candida* sp. (16 mm), and *Saccharomyces cerevisiae* (15 mm). The positive control used in this study was Ampicillin, and its maximum inhibitory zones against these fungal pathogens were 17–19 mm, 16–17 mm, and 14 mm, respectively.

The methanolic crude extract of *Hydropuntia* sp. also showed mild antibacterial properties against *Bacillus subtilis*, *Escherichia coli*, and *Vibrio cholera*. In contrast, it did not prevent the replication of *Staphylococcus epidermidis*, *Staphylococcus aureus*, and *Enterobacter cloacae* (Assaw et al., 2018). The moderate antibacterial activity of the crude extract could be attributed to the polarity index of the methanol solvent, which allowed the extraction of the bioactive compounds at ambient conditions. The extract was denser with a darker color, sticky, had no gelling appearance, had a lesser yield, and underwent 30 min of sonication in dimethyl sulfoxide (DMSO) before the bioassay (Assaw et al., 2018).

*H. edulis* has also been recognized as a good source of amino acids (glutamic acid, aspartic acid, glutamine, and alanine) and phytochemicals (terpenes, steroids, polyphenols, ketones, fucoxanthin, bromophenols, polyphloroglucinol). Priyadharshini et al. (2014) reported that *H. edulis* has several biomedical properties, such as antibacterial, antiviral, antifungal, antiprotozoal, anti-tumor, anti-inflammatory, antioxidant, hypoglycemic, and spasmolytic.

In a study, Mohanta et al. (2022) synthesized metallic nanoparticles using aqueous extract of *H. edulis.* The developed silver nanoparticles showed great potential against microbes and presented anticancer properties, which suggest their biomedical potential. Similarly, *H. edulis* extract has been used to produce zinc oxide nanoparticles and successfully tested for *in vitro* anticancer effects (Mohamed et al., 2019).

Additionally, *H. edulis* has been identified as a potential inhibitor of carbohydrate digestive enzymes. Thambi & Chakraborty (2023) supported the anti-hyperglycemic properties of *H. edulis.* Pyruvylated polysaccharide extract from *H. edulis* showed potential against hyperglycemia and anti-carbolytic activity. Additionally, once mixed with ethyl acetate, the methanolic extract inhibited the activity of α-amylase and α-glucosidase. These two enzymes play an important role in carbohydrate metabolism, as they digest carbohydrates. Hence, consuming *H. edulis*, with abundant bioactive compounds, could block enzyme activity, slow down the digestion of carbohydrates, and delay the glucose release into the blood in diabetics (Thambi & Chakraborty, 2023).

#### In agriculture and aquaculture

Seaweed has several benefits as a growth promoter in agriculture, including improved rooting systems, increased crop and fruit yield, enhanced photosynthetic activity, and strengthened plant resistance against microbial pathogens. Seaweeds commonly used in agriculture include *H. edulis* and *G. acerrosa*. *H. edulis* is a powerful biostimulant due to the macro and micro-minerals and other bioactive metabolites. Moreover, high fiber and organic matter in *H. edulis* also assist in holding moisture and minerals in soil (Mahusook et al., 2021). Bhushan et al. (2023) reported that *H. edulis* is a rich source of phytohormones and essential micro- and macro-elements. Indole-3-acetic acid (IAA) and the cytokinin (zeatin) were detected in the extract of *H. edulis*, which makes it great as a biostimulant (Mahusook et al., 2021). A study conducted in India has shown that a 10–33% yield improvement was recorded for the soybean crop when *H. edulis* was applied as a biofertilizer. Interestingly, the performance was better than applying the recommended dose of chemical fertilizers (Bhushan et al., 2023).

In the shrimp cultivation industry, the wastewater normally leads to eutrophication due to the high amount of nitrogen. This water environment could harm the growth of the shrimp. Baloo, Kikuchi & Azman (2011) evaluated the potential of *H. edulis* as a biofiltration agent in shrimp integrated systems. According to the results, *H. edulis* removed 70% of the ammonium, suggesting that it is an effective biofilter that improves water quality and encourages shrimp survival and growth. Table 3 depicts the application of *Hydropuntia* sp. in various food and non-food industries.

**Table 3:** *Hydropuntia* sp. application in various food and non-food industries.

**Industries**	**Species**	**Functional fraction**	**Application**	**References**
Food industries	*Gracilaria vermiculophylla*	Agar	Biodegradable agar film	Sousa et al. (2010), Yuguchi et al. (2016), Moldovan et al. (2017) and Othman et al. (2018)
	*Gracilaria* sp.	Seaweed pigment	Used as eco-friendly natural dyes	
	*Gracilaria* sp.	Agar	Making of Nori (dried edible seaweed)	
	*Gracilaria* sp.	Agar	Used of food grade agar as gelling agent, thickening and stabilizing agent in bakery, confectionery industry	
	*Hydropuntia edulis*	Food grade agar and protease	Thickening agent,agar strips, jellies, spreadable agar, and milk clotting protease	Arbita et al. (2020) and Bhushan et al. (2023)
	*Gracilaria cliftonii*	Food grade agar	Agar powder	Kumar & Fotedar (2009), Vuai & Mpatani (2019) and Xiao et al. (2021)
	*Gracilaria crassa*	Food grade agar	Agar powder	
	*Gracilaria salicornia*	Food grade agar	Agar powder	
	*Gracilaria lemaneiformis*	Food grade agar	Agar powder	
Agriculture	*Hydropuntia edulis*	Fresh biomass	Biostimulant for plants	Baloo, Kikuchi & Azman (2011) and Bhushan et al. (2023)
Aquaculture	*Hydropuntia edulis*	Agar	Phytoremediation agent in shrimp pond wastewater	
Pharmaceutical	*Hydropuntia edulis*	Fiber	Edible dietary fiber formulation as prebiotic	Debbarma et al. (2016) and Priyadharshini et al. (2014)
	*Hydropuntia edulis*	Extract	Cancer drug	
	*Hydropuntia edulis*	Extract	Antimicrobial agent	

The *Gracilaria* sp. has also been used as feed for shrimps. Hence, *H. edulis* may be an extractive species in Integrated Multi-Trophic Aquaculture (IMTA). IMTA is an aquaculture farming method incorporating species from different nutritional or trophic levels into the same system. This approach benefits the ecosystem in several ways, including economic diversification, ecological footprint reduction, managing the aquaculture waste release, and improving the social acceptability of the culture systems. The IMTA system in China incorporates three species of trophic levels, such as abalone (as herbivore), sea cucumber (as detritivore), and kelp (as primary producer). These three species formed a food web chain system where the abalone fed on kelp and produced organic waste and feed leftovers, which sea cucumbers can utilize. On the other hand, the kelp assimilates excretory products such as NH_4_ and CO_2_ that could be employed for growth and productivity. Zhang et al. (2018) suggested that *H. edulis* could also serve as the primary producer in IMTA. This could lead to socioeconomic diversification in society and the growth and development of *H. edulis* farming within IMTA systems and its applications across various industries.

## Concluding Remarks, Limitations, and Future Prospects

### Limitations and future remarks

Owing to the mounting demand for the health-promoting functional yet novel ingredients for foods and related applications, seaweeds such as *H. edulis* are considered a valuable resource. Seaweed cultivation is unique among farming systems, as it requires minimal inputs, unlike conventional agriculture, which typically depends on fertilizers, feed, and pesticides to achieve adequate yield. Therefore, due to the immense health-provoking implications, a unique set of beneficial fractions of seaweeds enabled them to be part of the daily diet in the European Union and other tropical regions (Healy et al., 2023). *H. edulis* has successfully been employed and evaluated as a prebiotic, antimicrobial, and biostimulation agent, besides other pharmaceutical benefits that started from the production of simple fractions (agar and pigments) and the complex metabolites (polyphenols, flavonoids, lignin, bioactive peptides, *etc.*) which are extracted from seaweed (Bhushan et al., 2023). Nevertheless, there is a promising opportunity to optimize the production of seaweed, which would ultimately augment the extraction and isolation of bioactive compounds with desired utility in food and pharmaceutical industries.

Most of the seaweeds are processed for the extraction and production of biopolymers and the derivation of certain extracts to be employed as supplements. These extracted polymers possess wider utility as food ingredients or in non-food applications such as cosmetics, pharmaceuticals, agriculture, and the paint industry.

However, relatively limited preference and acceptance have been granted to the raw seaweeds, including *H. edulis*, which could be due to marginal production, tedious processing, and possible safety concerns. As for this, establishing thorough regulations and standardization is needed to ensure quality attributes of *H. edulis* with no harmful residues, such as heavy metals and microbial pathogens. Additionally, the ensured availability of *H. edulis* would allow a consistent supply of raw material for extracting various functional ingredients, guaranteeing the increased utility and consumption of seaweed-based foods. Other than the conventional extraction of *H. edulis* agar, sophisticated green processing must be opted to optimize the consistent and high-quality production of bioactive compounds for enhanced usage in the functional food and pharmaceutical industries. Similar to other supplements, the safety of the *H. edulis*-derived ingredients must be evaluated through *in vitro* and *in vivo* experimentation, and the fate of the ingested *H. edulis*-based ingredients must be traced in the human body for possible harmful impacts on vital body parts, which is critical to unleashing the industrial and economic potential of this red seaweed.

### Conclusion

*H. edulis* is a seaweed that has the potential to be commercialized due to its diverse applications. Hence, the farming area of *H. edulis* should be enlarged to ensure a sufficient supply of seaweed for industrial purposes. It contains sufficient complex carbohydrates (agar), protein, polyphenols, vitamins, and minerals, making it a promising dietary supplement. Additionally, the bioactive substances in *H. edulis* are useful components for therapeutic applications. It has been successfully employed in numerous applications, including as a food ingredient, edible coatings and packaging in the food industry; a functional ingredient for health-promoting products; and as a bioremediant and biostimulant in aquaculture and agriculture. Consequently, the comprehensive study of *H. edulis* seeks to promote the enhanced cultivation of the species to produce agar and other bioactive compounds that could facilitate sustainable food, pharmaceutical, agriculture, and aquaculture industries.

*H. edulis*, a rich source of diverse bioactive compounds, could be a great candidate for developing supplements, nutraceuticals, and functional foods. Likewise, functional polysaccharides could allow the usage in developing exclusively biodegradable packaging films, coatings, hydrogels for food and medical applications. Additionally, a minimal input (fertilizer, *etc.*) requirement for its culture with simultaneous absorption of CO_2_ could make this seaweed a good contributor to blue carbon strategies and low-impact aquaculture for sustainable food systems. However, a comprehensive genomic and metabolic profiling of *H. edulis* could uncover massive functional bioactive compounds with greater pharmaceutical significance. Furthermore, the investigations about its impact on the human gut, immunity, and combating metabolic disorders could support its wider acceptability. Beyond the current applications of *H. edulis*, an interspecies comparative analysis and the bioactivities could be made to augment the seaweed significance as a sustainable food source. Moreover, integrating marine biology, aquaculture, food science, pharmacology, and environmental engineering could help to harness the complete potential of red seaweed for developing sustainable food systems.

## Additional Information and Declarations

### Data Availability

The following information was supplied regarding data availability: This is a literature review.
